# Research on the type and expansion mechanism of internal cracks in rocks under the action of PDC

**DOI:** 10.1038/s41598-025-09827-w

**Published:** 2025-07-11

**Authors:** Yu Liang, Boxin Du, Jianxi Ren, Shangxin Feng

**Affiliations:** School of Architecture and Civil Engineering, Xi’an University of Science and Technology, Xi’an, China

**Keywords:** Polycrystalline diamond compact (PDC), Crack propagation, Rock breaking mechanism, Digital image correlation (DIC), Liner cutting, Solid Earth sciences, Energy science and technology, Engineering

## Abstract

The rock fragmentation mechanism of polycrystalline diamond compact (PDC) constitutes a fundamental research focus in petroleum and mining engineering. This study presents an experimental investigation into crack propagation failure characteristics during cutter-rock interaction. Through an integrated experimental approach combining mechanical testing with digital image correlation (DIC) monitoring, the complete failure process from microcrack initiation to macroscopic fracture network development was quantitatively characterized. Experimental observations demonstrate significant dependence of crack propagation patterns on PDC cutter geometry, particularly the back rake angle. Initial fracture development (Stage I) exhibits predominant shear failure mechanisms, with tensile crack formation becoming progressively dominant during sustained loading (Stage II). The DIC-derived Displacement Field analysis revealed three distinct crack interaction modes: parallel propagation, bifurcation merging, and hierarchical networking. These findings provide a solid theoretical foundation for further research on PDC rock-breaking mechanisms, contributing to the optimization of drilling operations in engineering applications.

## Introduction

Polycrystalline Diamond Compact (PDC) bits have emerged as indispensable tools across a wide range of engineering applications, particularly in oil and gas exploration. Their exceptional cutting efficiency enables rapid penetration through complex geological formations, significantly enhancing drilling speeds, reducing operational cycles, and lowering extraction cost^1^. In unconventional resource development, such as shale gas extraction, PDC bits demonstrate remarkable adaptability to challenging formations, facilitating efficient resource recovery^2^. Additionally, their application in geological surveys provides critical insights into deep subsurface structures, aiding resource assessment and exploration^3^. Beyond hydrocarbon extraction, PDC bits are extensively utilized in water well drilling and mining operations, driving advancements in energy extraction and geological research due to their superior wear resistance and operational efficiency^4,5^. Consequently, a comprehensive understanding of rock fracture mechanisms and failure modes under PDC bit action is crucial for optimizing extraction efficiency, improving engineering design, and ensuring operational safety.

Significant progress has been made in understanding rock fracture under PDC bit action. Research has identified three primary crack modes: tensile, shear, and mixed-mode fractures. In brittle rocks, tensile cracks dominate, rapidly initiating and propagating under high-speed cutter penetration, while shear cracks prevail in ductile rocks^6^. However, the transition conditions between these crack modes remain poorly understood, particularly in relation to the microstructural heterogeneity of different rock types, highlighting the need for a unified theoretical framework. Crack morphology studies reveal diverse geometric patterns, including linear, curved, and branched forms, with a predominance of small-scale fractures^7^. Nevertheless, existing models struggle to accurately predict crack patterns and distributions in rocks with complex joint and fault systems, limiting their applicability in challenging geological conditions. Furthermore, while rock mechanical properties, cutter geometry, and loading conditions significantly influence crack propagation^8^real-time monitoring techniques and simulation algorithms face limitations in capturing the dynamic, multi-factor coupling effects prevalent in actual drilling operations.

In the study of rock failure modes under single-cutter PDC bit action, researchers have identified three primary mechanisms: tensile, shear, and mixed-mode failures. In brittle rocks, tensile cracks initiate and propagate rapidly due to low tensile strength, resulting in relatively smooth fracture surfaces^9^. In contrast, shear failures dominate in ductile rocks, characterized by irregular fracture surfaces and accompanied by plastic deformation^10^. Studies have also demonstrated the significant influence of loading rates and cutter geometry on failure mechanisms, with higher loading rates accelerating failure in brittle rocks and optimized cutter designs enhancing rock-breaking efficiency^11^. Despite these advancements, critical gaps remain. The effects of multi-factor coupling under complex geological conditions—such as high temperature, high pressure, and heterogeneous mineral compositions—are not fully understood, lacking systematic theoretical analysis and model development^12^. Furthermore, the dynamic evolution of failure modes across different rock types under varying loading conditions remains unclear, limiting the ability to predict mechanical responses during rock breaking. Additionally, the disparity between laboratory simulations and actual drilling conditions poses challenges in translating experimental findings to practical engineering applications^13^.

The analysis of internal crack morphology in rocks under single-cutter PDC bit action provides valuable insights into failure mechanisms^14^. Linear cracks perpendicular to the cutter force typically indicate tensile failure in brittle rocks, characterized by smooth fracture surfaces^15^. Conversely, curved or irregular cracks with pronounced branching are associated with shear failure in ductile rocks, often accompanied by plastic deformation^16^. Mixed-mode failures exhibit characteristics of both tensile and shear cracks^17,18^. This study aims to elucidate rock failure modes by examining internal crack morphology under single-cutter PDC bit action.

In rock mechanics research, Digital Image Correlation (DIC) has become an essential tool for investigating displacement and strain fields associated with internal crack propagation^19^. The non-contact measurement capabilities of DIC enable precise tracking of crack initiation and propagation, capturing even minute displacement changes during loading^20^. In brittle rocks, DIC has provided detailed observations of crack evolution, offering reliable data for understanding fracture mechanisms^21^. For ductile rocks, DIC has revealed complex strain distributions around crack tips, enhancing our understanding of deformation characteristics under various loading conditions^22^. The displacement and strain data obtained through DIC have facilitated the development of more accurate constitutive models that incorporate fracture mechanics, significantly improving the predictability of rock mechanical behavior and advancing theoretical frameworks in rock mechanics^23,24^.

By investigating internal crack morphology, utilizing DIC for strain field analysis, and examining failure modes under PDC bit action, this research provides fundamental insights into the mechanical behavior and failure mechanisms of rocks. These findings not only enhance the efficiency and quality of rock extraction but also offer theoretical foundations for engineering design and construction. Future research should focus on deepening our understanding of internal crack propagation mechanisms and developing more effective experimental methods and theoretical models to address the challenges in this field.

## Materials and methodology

### Experimental setups

**Fig. 1:**
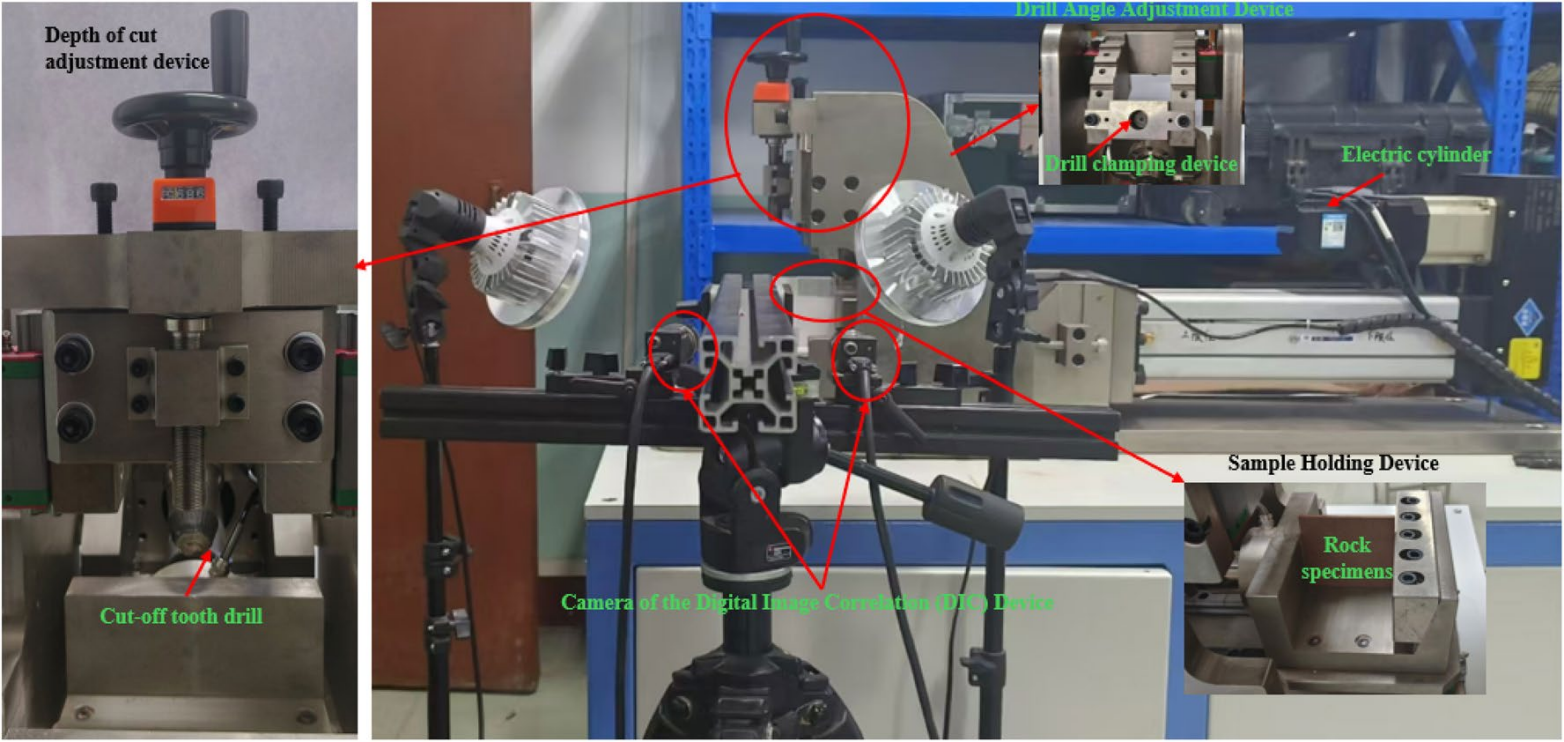
The diagram of the experimental set-up.

To investigate the crack extension pattern inside the rock under the action of PDC, a series of rock linear cutting tests were conducted on a custom-designed experimental platform (as shown in Fig. 1). As illustrated in the accompanying figure, the rock specimen is securely fixed in the clamping device of the testing apparatus. The drill bit holding mechanism is capable of precise vertical movement, enabling accurate control of the cutting depth within a range of 0–15 mm, with a resolution of 0.01 mm. Additionally, the platform facilitates horizontal movement to achieve linear cutting of the rock specimen.

The PDC cutter is mounted on the drill bit holding device, which is equipped with an angle adjustment mechanism to set the back rake angle of the cutter. The back rake angle can be adjusted within a range of 0–60°, with predefined settings at 0°, 15°, 30°, 45°, and 60°. Real-time monitoring of the cutting forces is achieved through a spoke-type tension load cell installed between the rock specimen clamping device and the force arm. The cutting experiments can be conducted in two control modes: force control, where the drill bit advances at a specified cutting force, and displacement control, where the drill bit operates based on predefined displacement targets and rates. This study primarily employs the displacement control mode to obtain the cutting force versus displacement curves during linear cutting, capturing real-time cutting forces.

The cutting process and crack propagation are recorded using Digital Image Correlation (DIC) technology, which provides detailed visualization of the cutting phenomena and crack distribution. This enables comprehensive analysis of the cutting process and crack evolution, as well as the strain development throughout the experiment. By analyzing the acquired data and images, the study aims to provide a clearer understanding of rock failure behavior and mechanisms under varying loading conditions, thereby enhancing the predictive accuracy of the proposed model.

**Fig. 2:**
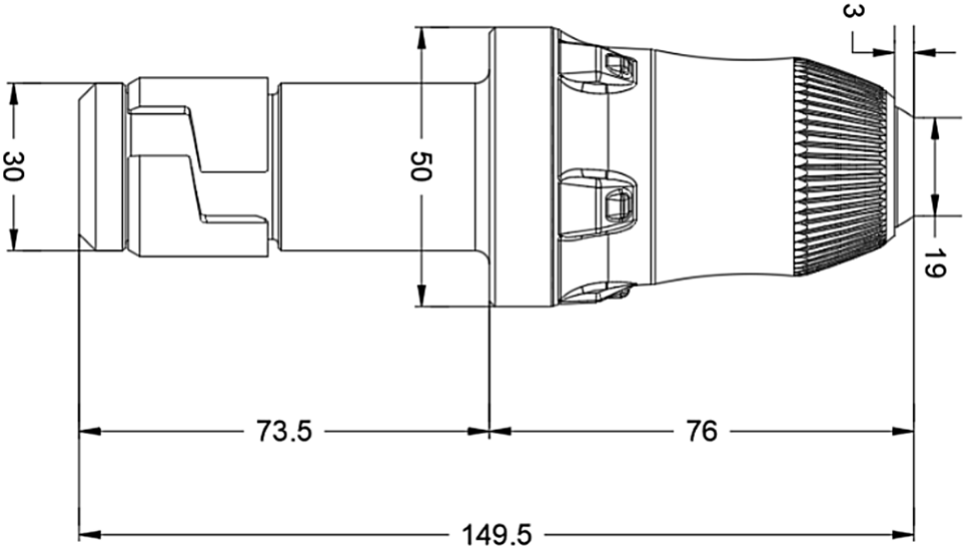
The shape and size of pick used in tests.

**Fig. 3:**
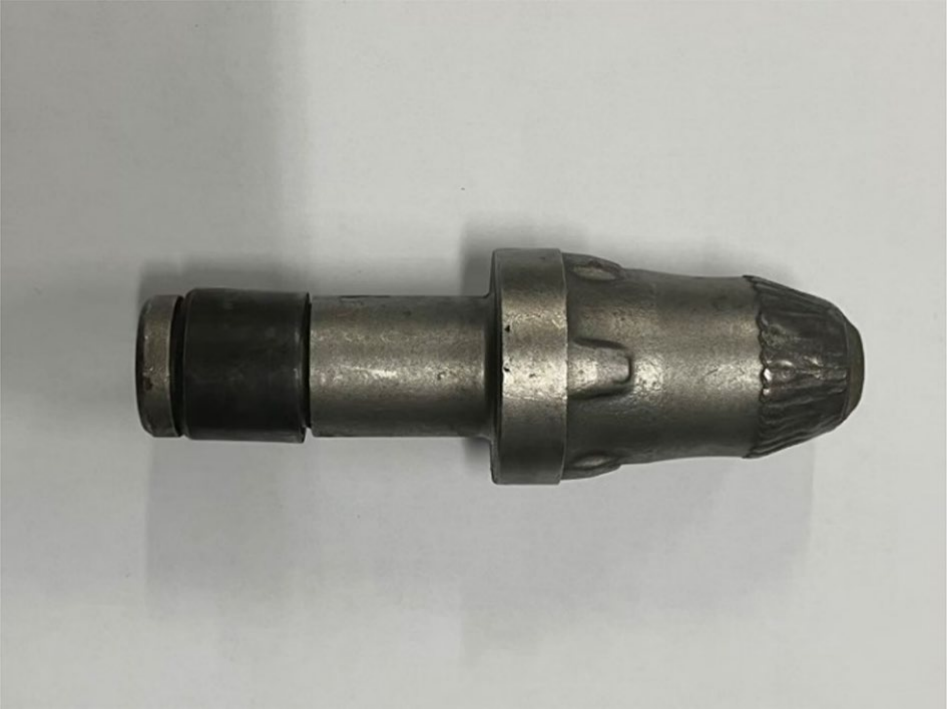
The physical image of pick used in tests.

A typical pick was used for tests, which consisted of pick body and a tip embedded in pick body. The pick body and embedded tip are made of cast iron and cemented carbide, respectively. The shape and size of pick are shown in Figs. 2 and 3.

**Fig. 4:**
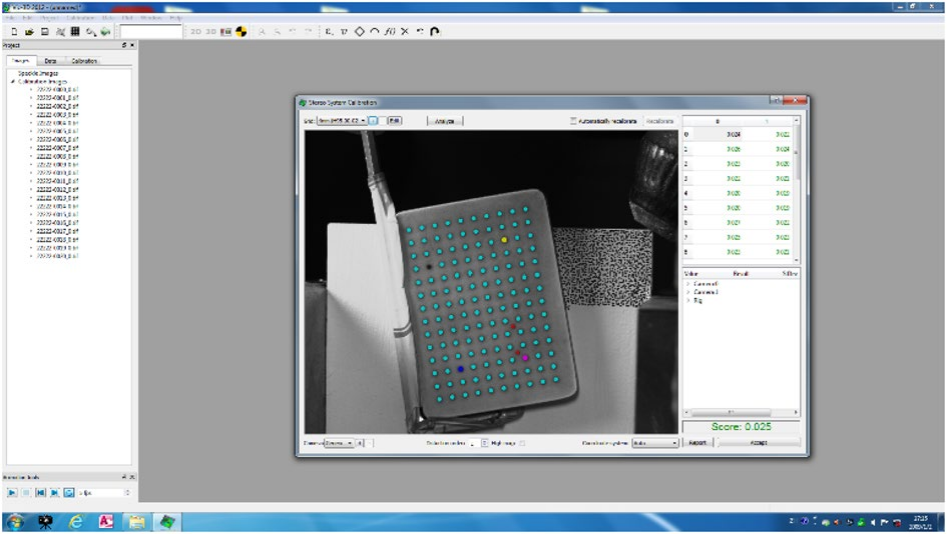
The Calibration process of DIC devices.

**Fig. 5:**
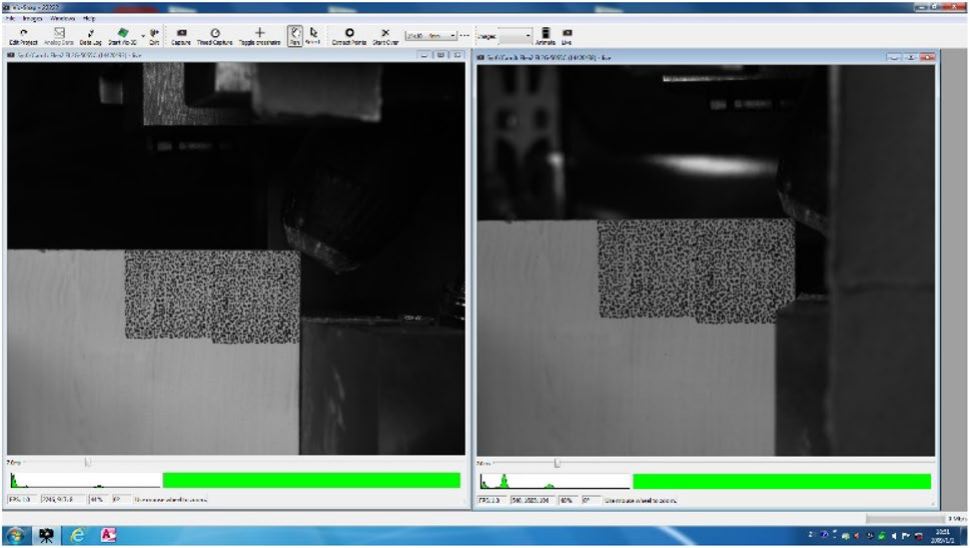
The Calibration results of DIC devices.

**Fig. 6:**
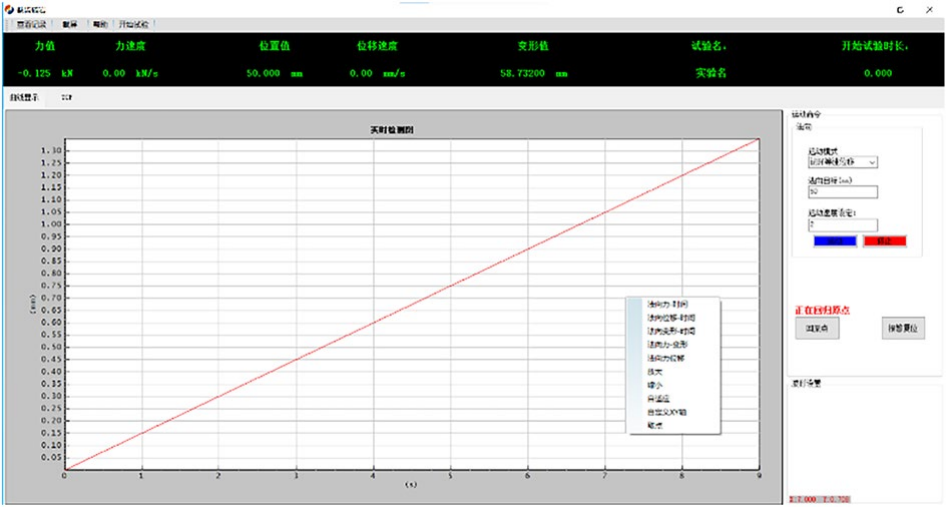
Graphical User Interface of the Experimental Platform Control Software.

**Fig. 7:**
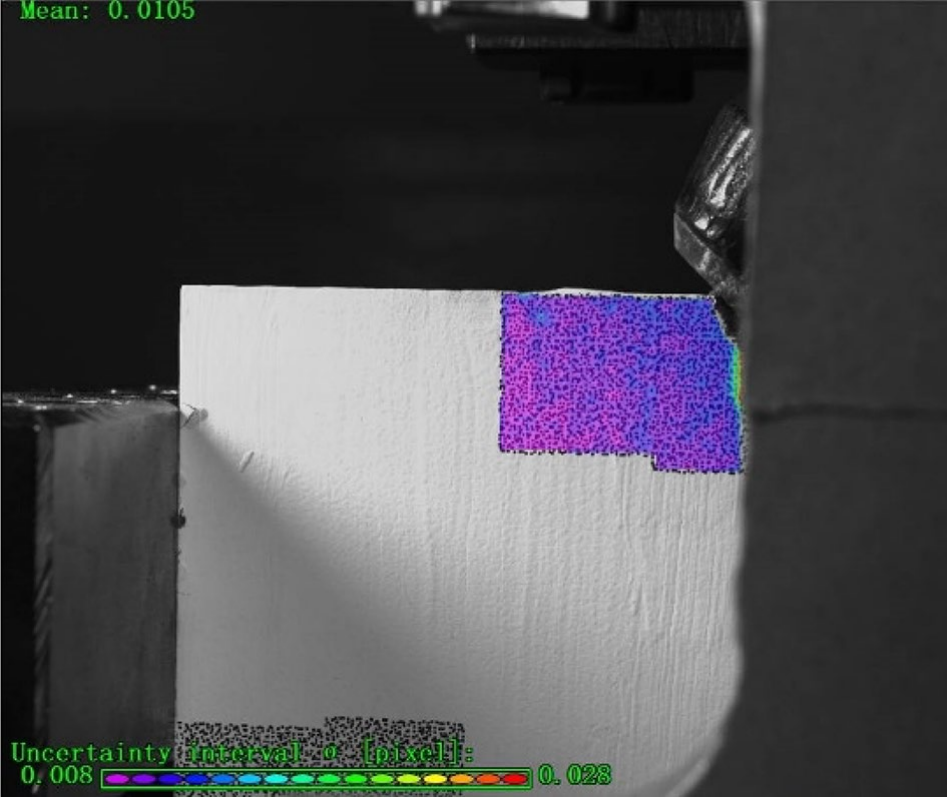
Digital Image Correlation (DIC) Device-Generated Cloud Maps during the Experimental Process.

The steps of this linear cutting tests were illustrated as below:

(1) Sample fixation: place the sample on the sample clamping device and fix it to ensure that the sample is firmly fixed to avoid displacement or shaking during the subsequent experiments.

(2) Installation of PDC drill bit: according to the required cutting angle, install the PDC drill bit in the corresponding position of the cutting teeth holder to fix it.

(3) Depth of cutting adjustment: by operating the depth of cutting adjustment device, the drill bit fixing device can move up and down precisely to control the cutting depth, with an adjustment range of 0–15 mm (with an accuracy of 0.01 mm).

(4) DIC device debugging and calibration: As shown in Figs. 4 and 5,adjust the position and focal length of the camera of the DIC device, according to the requirements of the experiment, adjusted in real time through the image feedback, until the image obtained by the camera is clear and complete, and then carry out the sample calibration to ensure that it meets the experimental data collection requirements.

(5) Starting the experiment: As shown in Figs. 6 and 7, set the cutting rate and cutting endpoint and other parameters in the control system, after confirming that there is no error, start the experiment. The rock specimen moves horizontally to the PDC drill bit at the set cutting speed along with the test bench.

### Rock specimen Preparation and experimental design

**Fig. 8:**
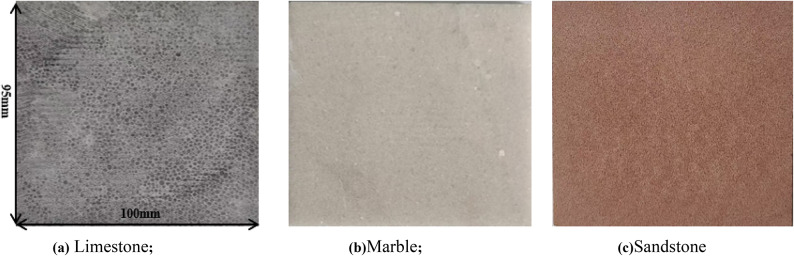
Rock samples.

For the cutting experiments, a Φ19 × 13 mm PDC cutter, commonly used in petroleum drilling applications, was employed. Three types of rocks—limestone, marble, and sandstone—were selected based on their homogeneity, isotropy, and absence of visible pre-existing fractures. These rock specimens were meticulously machined into blocks measuring 100 × 95 × 5 mm (Fig. 8). To ensure experimental consistency, the surfaces of the blocks were polished to a high finish, and strict tolerances were maintained for the parallelism of opposing faces. This preparation process guarantees the reliability and repeatability of the experimental results, providing a robust foundation for analyzing the cutting mechanics and fracture behavior under PDC cutter action.

The experimental design focuses on investigating the influence of rock type and cutter geometry on cutting forces, crack propagation, and failure mechanisms. The selection of limestone, marble, and sandstone allows for a comparative analysis of rock behavior across a range of mechanical properties, including hardness, brittleness, and grain structure. The polished surfaces facilitate accurate strain measurement and crack observation using DIC technology, while the precise dimensional control ensures uniform loading conditions during the cutting tests. This systematic approach enables a comprehensive understanding of the interaction between PDC cutters and different rock types, contributing to the optimization of drilling operations in various geological settings.

The physical and mechanical properties of these rocks are shown in Table 1:

**Table 1:** Basic physical and mechanical properties of experimental rocks.

Rock species	ρ (g/cm^3^)	E (GPa)	µ (1)	C(MPa)	φ(°)	UCS(MPa)
Limestone	2.60	35.42	0.29	38.12	54.12	165
Marble	2.49	17.38	0.31	24.28	47.90	95.3
Sandstone	2.73	47.65	0.16	28.72	53.52	114

In this experiment, parameters such as cutting depth, rake angle, and rock type were changed, and repeated tests were conducted. The parameter design of the experiment is shown in Table 2.

**Table 2:** The parameter design in cutting test.

Depth/mm Anteversion angle/°	1	2	4	6
30	Limestone; Marble; Sandstone			
45				
60				

### Fundamentals of DIC (Digital image Correlation) technology

**Fig. 9:**
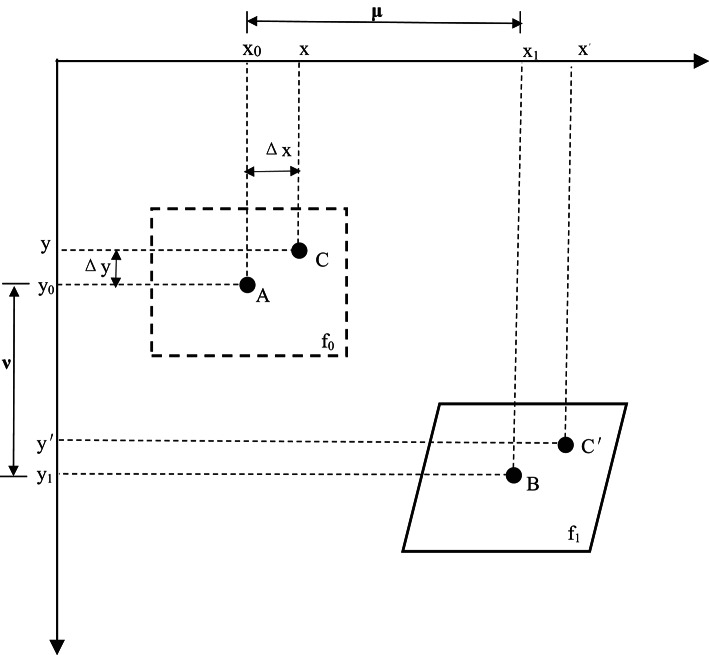
Analysis Domain of Digital Image Correlation (DIC) Method.

DIC integrates conventional digital photography with civil engineering measurement techniques to accurately quantify structural surface deformations. By capturing precisely aligned and localized images, DIC enables the comparison of measurements and identification of displacement variations, as illustrated in the accompanying figure. The technique employs high-speed cameras to track the deformation of a region centered at point A (x₀, y₀), denoted as f₀, to its deformed state centered at point B (x₁, y₁), denoted as f₁ (Fig. 9). Within the region f₀, a point C (x, y) undergoes displacement to point C′ (x′, y′) in the deformed region f₁. The relationship between these points is described by the following transformation equations:

1 $${x^{'}} = x + u + \frac{{\partial u}}{{\partial x}}\Delta x + \frac{{\partial u}}{{\partial y}}\Delta y$$

2 $${y^{'}}=y+v+\frac{{\partial v}}{{\partial x}}\Delta x+\frac{{\partial v}}{{\partial y}}\Delta y$$

This formulation allows for precise quantification of strain fields and deformation patterns, providing critical insights into the mechanical behavior of materials under load. The high-resolution imaging and advanced correlation algorithms employed in DIC ensure accurate measurement of both small- and large-scale deformations, making it an indispensable tool for experimental mechanics and structural analysis.

By leveraging DIC, this study captures the full-field displacement and strain evolution during rock cutting, enabling a detailed analysis of crack initiation, propagation, and failure mechanisms. The technique’s non-contact nature and high spatial resolution make it particularly suitable for studying brittle and heterogeneous materials, such as rocks, under dynamic loading conditions. The resulting data provide a deeper understanding of the complex interactions between PDC cutters and rock formations.

## Crack propagation and failure modes

### Crack type identification and classification

**Fig. 10:**
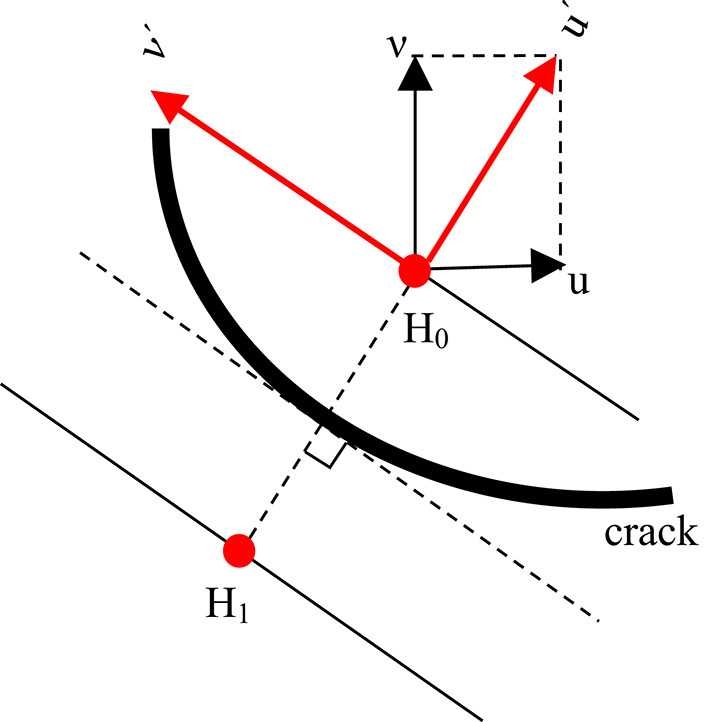
Mechanical Analysis of Sites on.

**Fig. 11:**
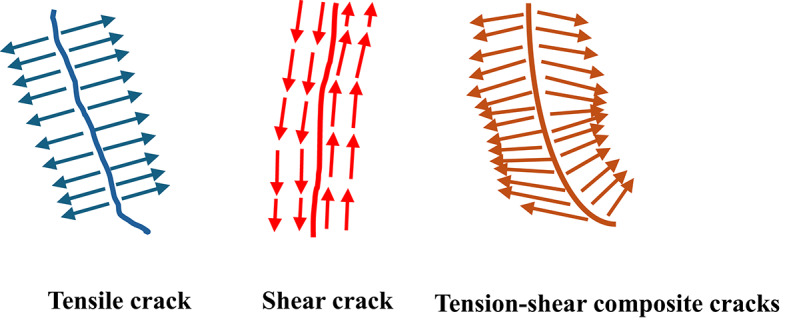
Mechanical Models of Different Types of Cracks.

To investigate the characteristics of internal cracks in rocks under PDC cutter action, a systematic analysis was conducted by selecting multiple pairs of symmetric points, H₁ and H₀, on either side of the crack. The displacements of these points were transformed from the original u-ν coordinate system to a local u′-ν′ coordinate system, as illustrated in Fig. 10. Based on the u′ and ν′ displacement components of points H₁ and H₀, the cracks were classified into three distinct types, as depicted in Fig. 11:

3 $${u^{'}}=u \cdot \sin \beta +v \cdot \cos \beta$$

4 $${v^{'}}=u \cdot \sin \beta +v \cdot \sin \beta$$

1. Tensile Cracks (Mode I): When the u′ components of the symmetric points exhibit opposite signs (one positive and one negative), it indicates that the points on either side of the crack are moving away from each other in a direction perpendicular to the crack. This behavior is characteristic of tensile cracks (Mode I).
2. Shear Cracks (Mode II): If the difference in the ν′ components of points H₁ and H₀ exceeds a threshold value of 0.01, it signifies that the points on either side of the crack are undergoing relative motion in opposite directions parallel to the crack. This behavior is indicative of shear cracks (Mode II).
3. Mixed-Mode Cracks (Mode I-II): When the u′ components of the symmetric points show opposite signs and the difference in the ν′ components exceeds 0.01, the crack is classified as a mixed-mode crack (Mode I-II), exhibiting both tensile and shear characteristics.

By analyzing the displacement patterns of symmetric points, this methodology enables precise classification of crack types, providing critical insights into the mechanisms of crack propagation within rocks. This approach not only enhances the understanding of rock fracture behavior under PDC cutter action but also serves as a robust foundation for developing predictive models and optimizing drilling operations in various geological settings. The results of this analysis offering a quantitative framework for characterizing complex fracture patterns and their evolution under mechanical loading.

### Crack propagation processes and morphological evolution

**Fig. 12:**
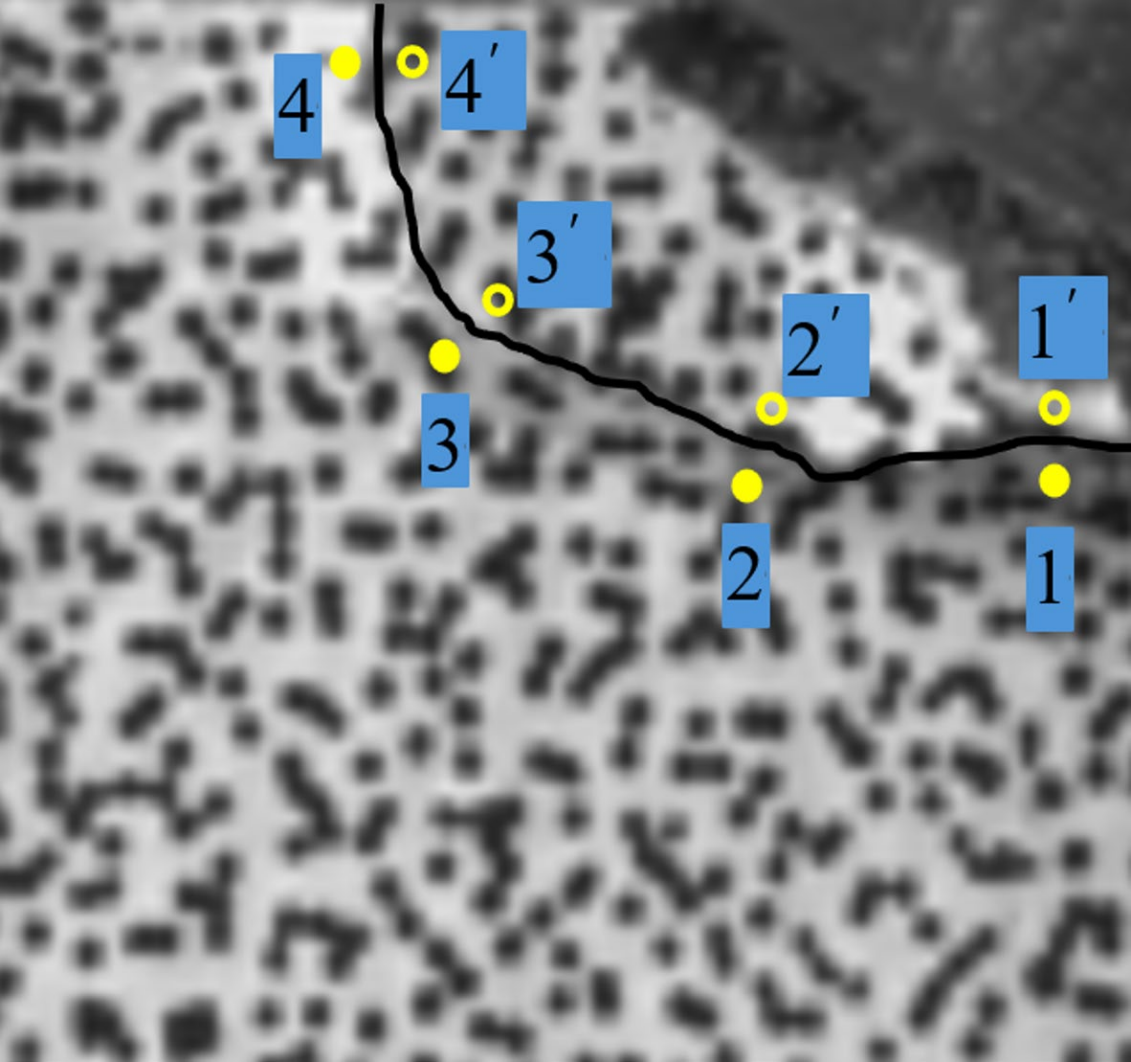
Location of crack analysis points.

Figure 12 illustrates the crack propagation patterns observed during the cutting process with a back rake angle (α) of 45°. Given the relatively small scale of the cracks, four pairs of symmetric points were selected on either side of each crack for detailed analysis. This approach ensures a comprehensive evaluation of the displacement and strain fields associated with crack initiation and growth. The symmetric point analysis provides critical insights into the local deformation behavior, enabling the classification of crack types (Mode I, Mode II, or mixed-mode) and the quantification of crack opening and sliding displacements. By examining the displacement trends of these symmetric points, the study elucidates the influence of cutter geometry on crack propagation mechanisms, contributing to a deeper understanding of rock fracture dynamics under PDC cutter action.

**Fig. 13:**
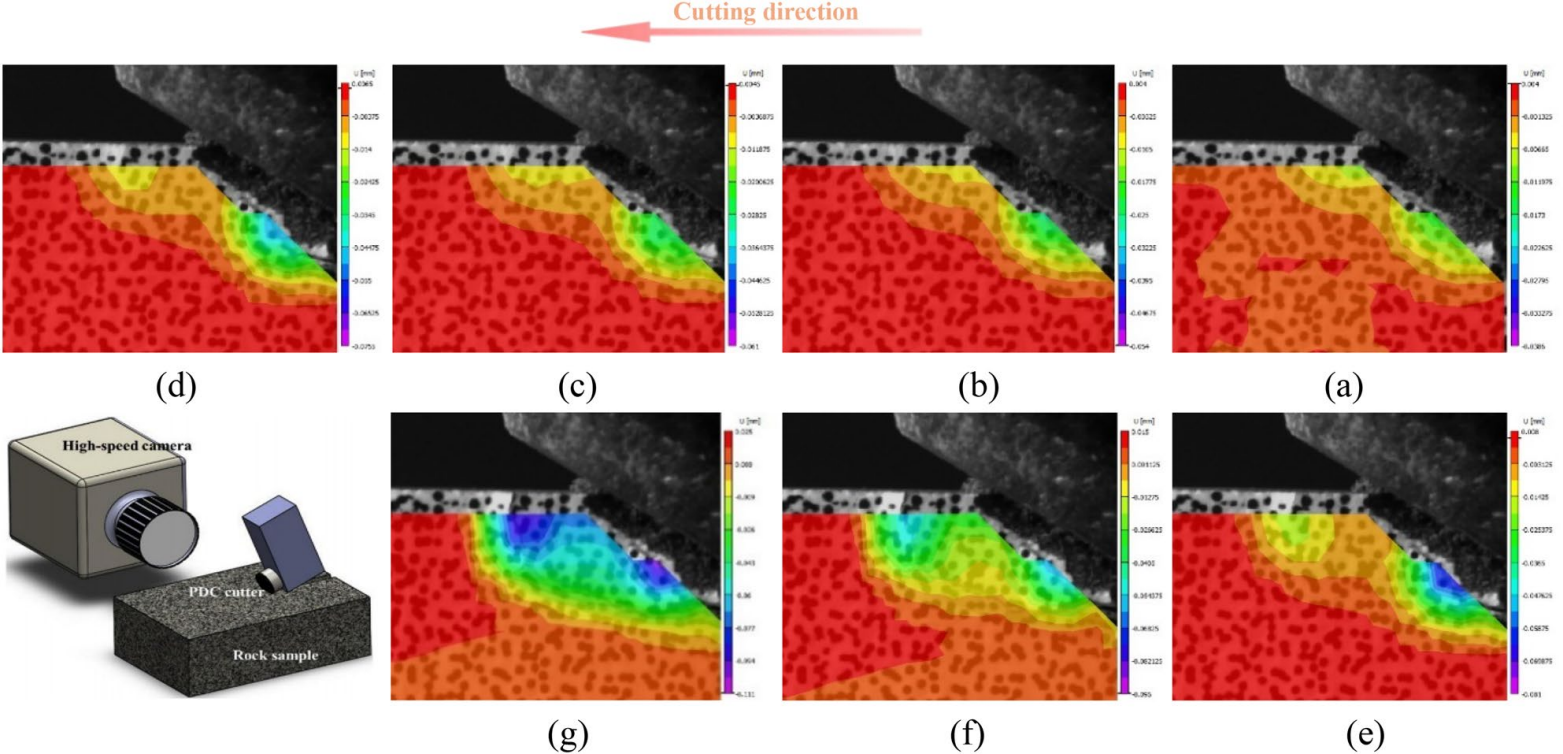
Evolution of DIC Displacement Field and Crack Initiation during Chip Formation.

Figure 13 presents a systematic observation of crack evolution mechanisms during rock cutting, utilizing displacement field analysis based on Digital Image Correlation (DIC) technology (what is shown in Fig. 13 is not complete specimen, but a selected analysis region for Digital Image Correlation (DIC) analysis).

Digital Image Correlation (DIC) cloud maps in rock cutting process reflect the evolution of displacement field. At the initial stage of cutting, the rock material in front of the cutter undergoes shear deformation. The DIC cloud map shows a significant shear displacement concentration zone, and cracks are initiated due to shear displacement. As the cutting progresses, with the expansion of cracks, a small amount of tensile displacement appears at the crack tip and surrounding areas. However, throughout the process, shear displacement is dominant, which can be seen from the large - scale distribution of shear - related displacement patterns in the cloud maps.

From the perspective of stress, the initial shear displacement is caused by shear stress. When the shear stress exceeds the rock’s shear strength, cracks start to form. In the subsequent development, although tensile stress is generated due to the change of displacement field (resulting in tensile displacement), the overall stress state is still dominated by shear stress. The distribution and evolution of displacement in the DIC cloud maps also verify that the shear stress plays a leading role in driving crack generation and development, and the tensile stress only has a secondary and local influence.

In general, the DIC cloud maps clearly show that in rock cutting, crack generation and development are mainly under the action of shear - dominated displacement and stress, with tensile - related factors playing a supplementary role.

**Fig. 14:**
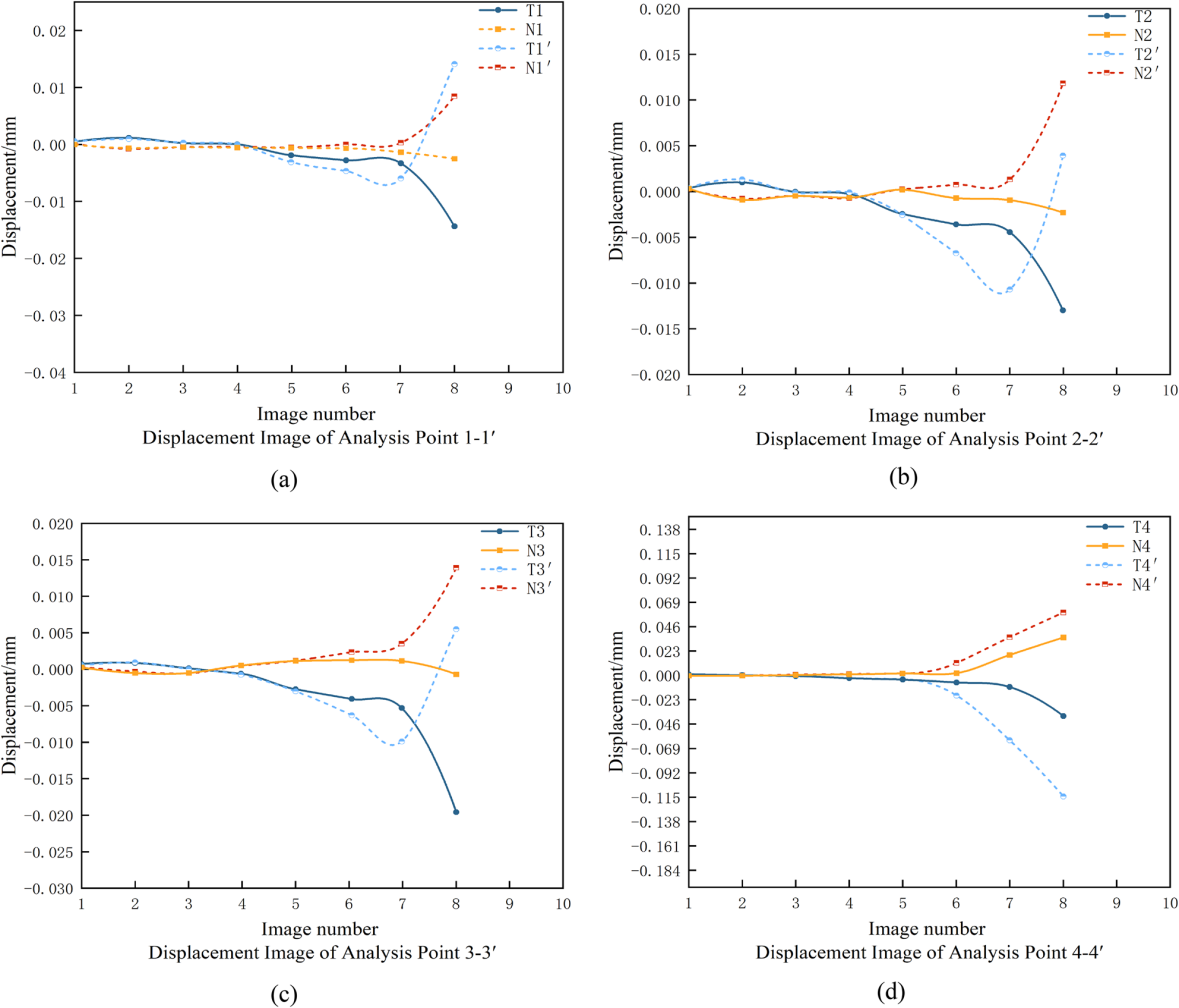
Plot of normal and tangential displacements at symmetry points on both sides of the crack(α=45°).

As depicted in Fig. 14, the DIC system precisely monitors the normal (N) and tangential (T) displacements of symmetric points on either side of internal cracks within the rock mass under the action of a single PDC cutter. The crack evolution can be divided into two distinct phases based on the displacement characteristics:

4. Pure Shear Phase (Initial Stage): During the initial phase, the tangential displacement difference increases rapidly, indicating significant relative sliding between the rock masses on either side of the crack due to the shear force exerted by the cutter. In contrast, the normal displacement difference remains minimal, suggesting that the crack propagation is predominantly governed by shear stress, exhibiting a pure shear mode.
5. Tensile-Shear Coupling Phase (Progressive Stage): As the cutter continues to act on the rock, the crack transitions into a tensile-shear coupling phase. Here, the normal displacement difference begins to increase significantly, reflecting the growing influence of tensile stress and the tendency for the crack surfaces to separate. Simultaneously, the tangential displacement difference remains substantial and continues to evolve, indicating that shear stress still plays a critical role. As shown in Fig. 14 d, shear failure remains dominant during this phase, but the crack morphology becomes more complex, combining both tensile and shear characteristics.

By comparing the normal and tangential displacement data of symmetric points at the same moment, the dynamic variation trend of the displacement difference between the two types can be clearly deduced. The analysis of displacement differences leads to the following conclusions: By monitoring the normal and tangential displacement differences using DIC, the transition from pure shear to tensile-shear coupling can be clearly captured. Under the action of a single PDC cutter, rock cracks initially propagate in a pure shear mode before transitioning to a tensile-shear coupling phase. This evolution from a simple shear-dominated morphology to a complex mixed-mode morphology provides critical insights into the rock-breaking mechanism. These findings enhance our understanding of the fracture dynamics in rocks under PDC.

### Internal crack morphology variations

**Fig. 15:**
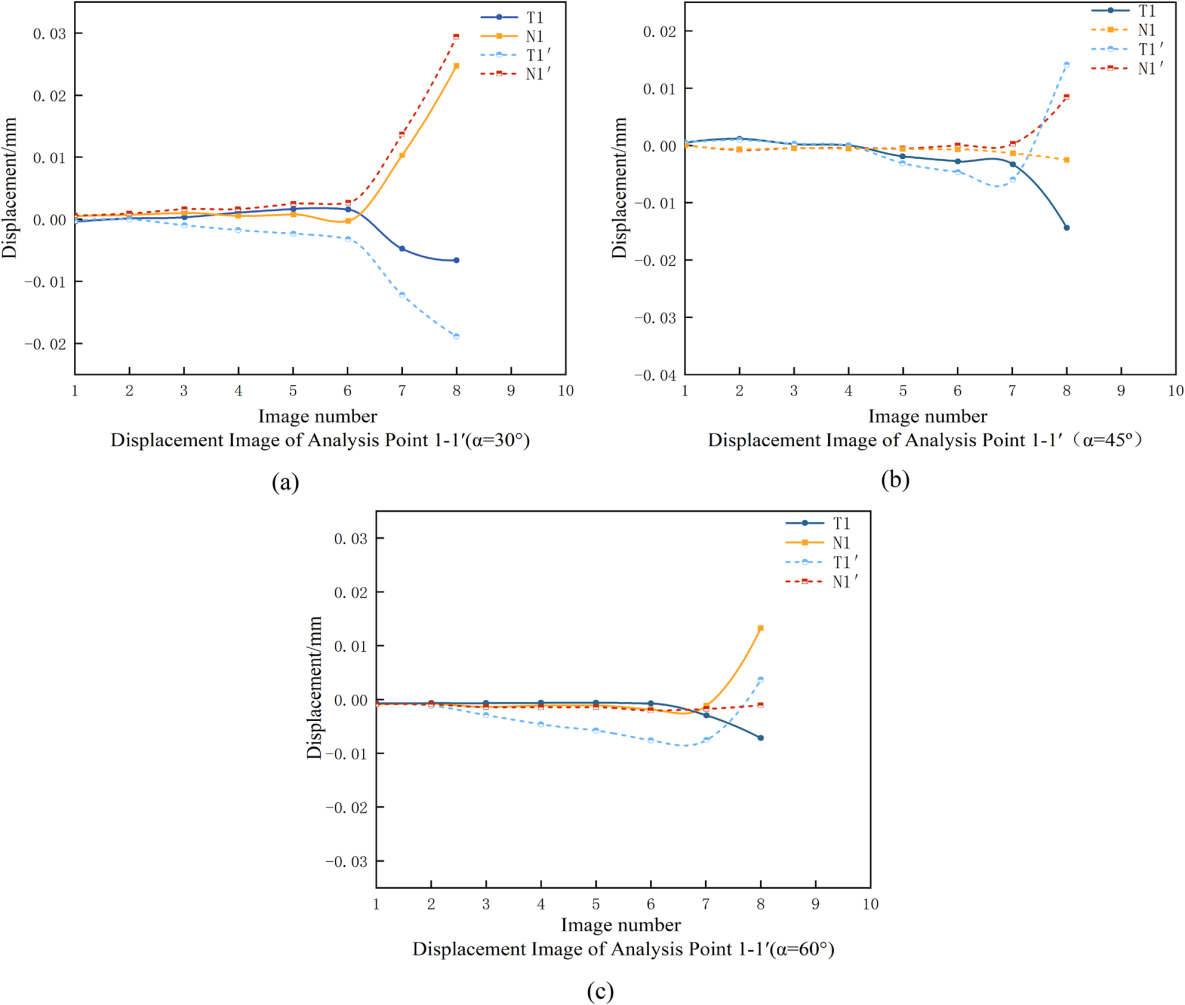
Normal and tangential displacements of the symmetry point of the crack tip at different angles.

As illustrated in Fig. 15(a), when the back rake angle (α) of the PDC cutter is set to 30°, both tangential and normal displacement differences are observed at the crack initiation point. According to fracture mechanics principles, the tangential displacement difference indicates the presence of shear stress, while the normal displacement difference signifies tensile stress. This suggests that, at a back rake angle of 30°, crack initiation is driven by a combination of shear and tensile stresses.

In contrast, as shown in Fig. 15(b) and 15(c), for back rake angles of 45° and 60°, only tangential displacement differences are detected at the crack initiation point, implying that crack formation is primarily induced by shear stress. This observation highlights a significant shift in the stress state and failure mechanism as the back rake angle increases. Specifically, the rock failure mode transitions from a mixed stress state (shear and tensile) to a shear-dominated failure mechanism.

These findings demonstrate that the back rake angle plays a critical role in determining the stress distribution and failure behavior of rocks under PDC cutter action. At lower back rake angles, the interaction between shear and tensile stresses leads to complex crack initiation and propagation, while higher back rake angles promote shear-dominated failure.

**Fig. 16:**
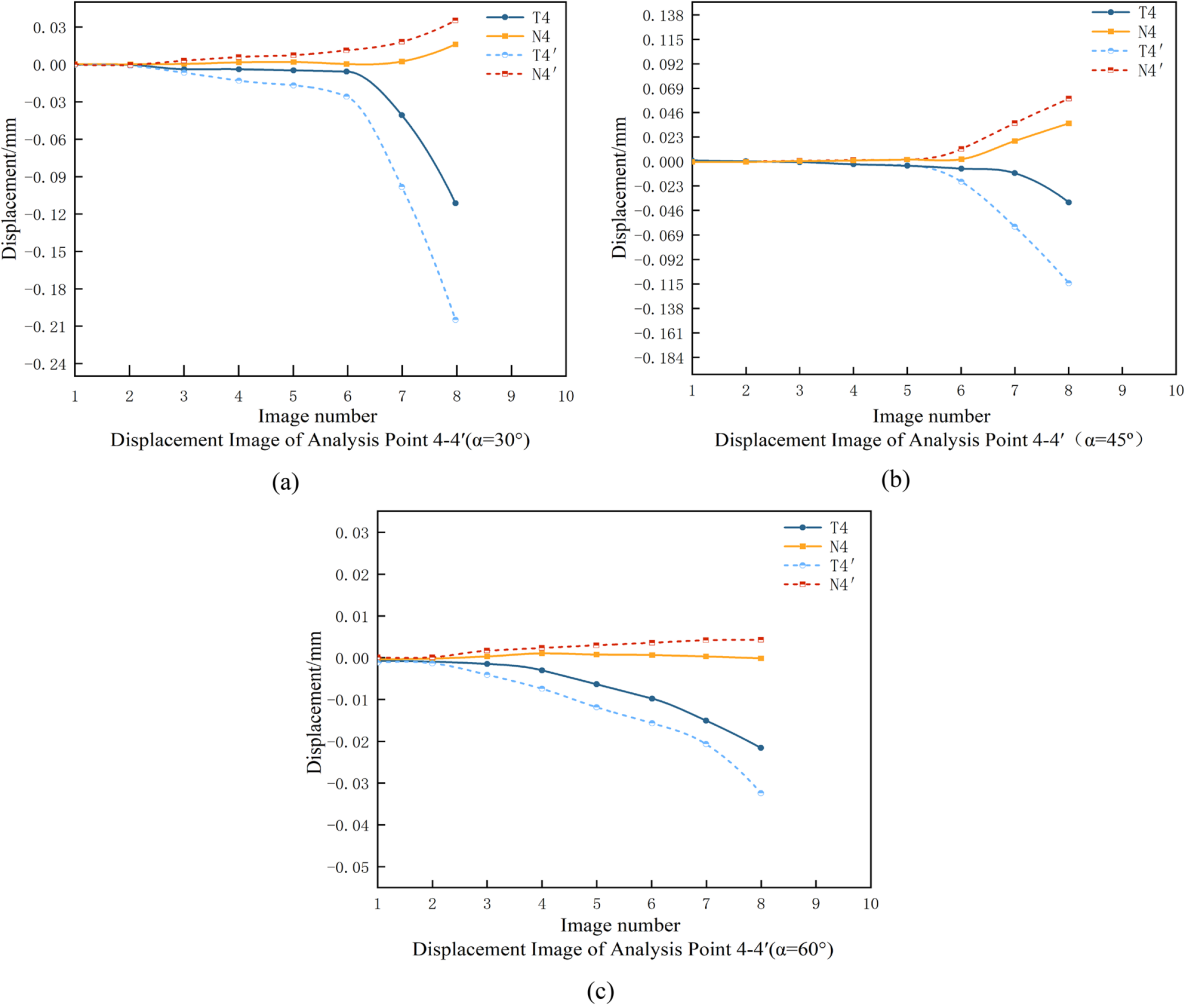
Normal and tangential displacements of the symmetry point at the end of the crack at different angles.

Figure 16 presents the normal and tangential displacements of symmetric points at the crack tip under three different back rake angles (α). A clear trend emerges from the comparative analysis of the data in Fig. 16(a), 16(b), and 16(c): as the back rake angle increases, the ratio of tangential to normal displacement differences at the crack tip also increases. This trend indicates that the influence of shear stress becomes progressively more dominant relative to tensile stress during crack initiation and propagation at the crack tip.

The increasing ratio of tangential to normal displacement differences aligns closely with the observations in Fig. 15, further validating that shear stress plays a more critical role in rock failure as the back rake angle increases. These results highlight the significant impact of cutter geometry on the stress state and failure mechanisms in rocks. Specifically, higher back rake angles promote shear-dominated failure, while lower angles result in a more balanced contribution of shear and tensile stresses.

This study provides critical insights into the relationship between back rake angle, stress distribution, and crack propagation behavior. The findings underscore the importance of optimizing cutter geometry to control fracture patterns and enhance drilling efficiency in various geological conditions.

## Conclusions

This study employs Digital Image Correlation (DIC) technology to investigate the rock failure process. Through detailed analysis of rock surface images, a novel diagnostic methodology for fracture mode identification is developed through kinematic analysis of symmetric point displacement vectors. Experimental data analysis reveals that coexisting tensile (Mode I) and shear (Mode II) fracture trajectories, confirming a mixed-mode I/II fracture mechanism during liner cutting. Quantitative stress field reconstruction demonstrates progressive transformation of rock’s internal stress state: initial triaxial tension-shear coupling evolves into shear-dominant stress configuration with increased cutter penetration depth. Mechanistic analysis establishes shear stress dominance in crack nucleation processes under liner cutting. The critical phase of crack initiation exhibits localized shear stress intensification, triggering rapid fracture nucleation within cutter-proximal zones. While tensile stress components facilitate crack opening during propagation phase, shear stress redistribution governs three essential aspects of fracture dynamics. These findings provide a comprehensive understanding of rock failure mechanisms under PDC cutter, offering valuable insights for optimizing cutter design and drilling operations.

## Data Availability

Data Availability StatementThe datasets used and/or analysed during the current study available from the corresponding author on reasonable request.Sincerely! Boxin Du23204228102@stu.xust.edu.cn.
